# Design and predictive modeling of a veterinary drug detection sensor in paddy field water based on artificial neural networks

**DOI:** 10.1038/s41598-026-38752-9

**Published:** 2026-02-13

**Authors:** Junshi Huang, Bolin Huang, Shuanggen Huang, Xiaobin Wang, Jinhui Zhao

**Affiliations:** 1 Key Laboratory of Modern Agricultural Equipment of Jiangxi, Jiangxi Agricultural University, Nanchang, 330045 China; 2 School of Physics and Electronic Information, Nanchang Normal University, Nanchang, 330045 China

**Keywords:** Veterinary drugs, Interdigitated electrode, Model migration, Artificial neural networks, Chemistry, Engineering

## Abstract

For rapid real-time detection of veterinary drug residues in paddy field water, we developed a novel sensor system using interdigitated electrodes as detection probes and the STM32F405RGT6 microcontroller as the core processing unit. The hardware architecture integrates multiple functional modules including excitation signal generation, signal detection, signal processing, LoRa coupled with 4G wireless communication, voltage regulation, and lithium battery charging. The system acquires three types of measurement data (amplitude ratio, phase difference, and their combination) from water samples containing sulfamethazine, ofloxacin, doxycycline hydrochloride and tetracycline hydrochloride across a broad frequency spectrum from 200 Hz to 100 MHz. Through Competitive Adaptive Reweighted Sampling (CARS) for feature selection and artificial neural network modeling, we established a multi-input multi-output concentration prediction model. Comparative analysis demonstrated superior performance when using phase difference data as model input, achieving prediction coefficients of determination (R^2^) between 0.7831 and 0.8713 with root mean square errors of prediction (RMSEP) ranging from 22.0759 to 28.1526 mg/L. Studies showed that this sensor device could effectively detect the contents of four veterinary drugs, namely sulfamethazine, doxycycline hydrochloride, ofloxacin, and tetracycline hydrochloride, in paddy field water, thus realizing the rapid and real-time monitoring of veterinary drugs in paddy field water.

## Introduction

The sources of veterinary drug residues in water bodies are remarkably extensive and diverse, mainly involving three core fields: animal husbandry, aquaculture, and the pharmaceutical industry. In animal husbandry production, farmers need to routinely use various veterinary drugs to prevent and treat animal diseases. However, some of the veterinary drugs ingested by livestock and poultry are not completely metabolized, and their prototypes and metabolites will be discharged out of the body along with feces and urine^1,2^. Most of these excrements are directly used as organic fertilizers in farmlands, or discharged into the environment without standardized treatment, and eventually flow into water bodies through surface runoff, soil infiltration and other pathways. In aquaculture, to control diseases of cultured organisms and improve breeding survival rates, drugs are often directly added to the aquaculture water. If the drug-containing aquaculture wastewater is directly discharged without effective treatment, it will cause direct pollution to natural water bodies^3,4^. In the veterinary pharmaceutical industry, industrial wastewater containing pharmaceutical ingredients is generated during the production and processing processes. If enterprises fail to take strict wastewater treatment measures, the residual veterinary drugs in the wastewater will be directly discharged into the aquatic environment, becoming an important source of water pollution^5,6^. Veterinary drug residues that enter the aquatic environment can migrate to rivers with water flow, and then enter paddy fields through irrigation canals and other channels, resulting in the pollution of paddy field water bodies^7,8^ .Veterinary drugs in paddy water pose significant risks to ecological safety and could also affect human health through the food chain. Their main hazards are as following: residues of veterinary drugs in paddy water not only pose a serious threat to the safety of rice consumption but could also further endanger human life and health through the food chain; If veterinary drugs persisted in the rice growth environment for a long time, they may stimulate pathogenic microorganisms to undergo genetic mutations, thereby enhancing their resistance to veterinary drugs^9–11^; The accumulation of veterinary drugs in paddy soil could affect soil fertility and disrupts microecological balance^12–14^; Veterinary drugs also have a certain impact on the activity of paddy water. Therefore, the detection and control technologies for veterinary drugs in water bodies are of great significance for human life and health, the quality and safety of rice and ecological construction.

To date, research on methods for detecting veterinary drug content has come a long way, with many methods developed and still under development^15–18^. The earliest methods for detecting drugs, especially veterinary drugs, were biological methods, which were time-consuming and had low sensitivity. Subsequently, modern techniques, such as gas chromatography, high-performance liquid chromatography and mass spectrometry (including chromatography-mass spectrometry) emerged one after another. At the cutting edge of analytical chemistry, these methods has been extensively applied in the realm of environmental monitoring, yielding a multitude of significant achievements and paving the way for the detection of organic pollutants in the environment. The main methods for detecting veterinary drug content include: immunoassay method, capillary electrophoresis, gas chromatography-mass spectrometry, liquid chromatography-mass spectrometry, high-performance liquid chromatography-ultraviolet detection and high-performance liquid chromatography-fluorescence detection^19,20^. Based on these methods, related research has been conducted both domestically and internationally. Simultaneous determination of 22 antibiotics in aquaculture sediments was achieved using pass-through solid-phase extraction coupled with ultra-high performance liquid chromatography-tandem mass spectrometry (UPLC-MS/MS). In the concentration range of 2.00 to 100 ug/L, the 22 antibiotics exhibited good linearity, with correlation coefficients (r) all greater than 0.998. At three spiked levels (10, 50, and 100 μg/kg), the recoveries ranged from 60.3 to 109.7% with six replicates, and the relative standard deviations were between 1.2% and 13.2%^21^. Additionally, capillary electrophoresis was employed for the simultaneous separation and detection of multiple classes of antibiotics in water. Based on the peak area calculations of antibiotics such as ofloxacin (OFL), the relative standard deviation (RSD, n = 5) and detection limit for OFL were 2.6% and 0.09 mg/L, respectively^22^. Development of a fluorescence polarization immunoassay based on a specific single-chain antibody for the detection of 13 sulfonamide residues in milk, with detection limits ranging from 2.74 to 20.36 μg/L^23^.

However, although the aforementioned veterinary drug detection methods can achieve high-precision quantitative analysis of veterinary drug content, they generally suffer from such drawbacks as cumbersome pre-detection sample preparation procedures, complicated operation during the detection process, and a long overall detection time of several hours. In addition, the high cost of related detection equipment makes it difficult to meet the practical needs of grassroots popularization and on-site rapid detection.o address this technical bottleneck, this study proposes the use of the dielectric property method for veterinary drug detection—this method eliminates the need for complex sample pretreatment procedures, enables the completion of detection within 4–6 min, and allows for on-site real-time analysis via portable devices, thus effectively overcoming the limitations of traditional methods. In addition, dielectric property detection technology also boasts prominent advantages such as rapidity, high efficiency, non-destructiveness, environmental friendliness, reliability and accuracy, thus attracting extensive attention and in-depth research from scholars at home and abroad^24^.

The engineering application of dielectric property detection technology has accumulated abundant cases in multiple fields, providing practical references for its expansion into veterinary drug detection scenarios. For example, Ren Tong designed a dielectric parameter measurement device for straw compost, conducted dielectric parameter detection on sheep manure-corn straw compost samples, and constructed a support vector regression (SVR) model by extracting characteristic variables and combining full-frequency dielectric parameters, achieving accurate prediction of compost moisture content with the model’s coefficient of determination (R^2^) as high as 0.9877^25^. Lü Liming developed a prototype for non-destructive detection of internal quality of apples based on dielectric characteristics; through comparative tests of multi-frequency dielectric parameters and systematic error analysis, the device was verified to have stable capability in measuring apple dielectric parameters at low, medium and high frequency bands, with the overall detection accuracy exceeding 95%^26^. Guo Wenchuan et al. collected dielectric parameter spectrum data of Fuji apples at 101 frequency points within the frequency range of 10 to 4500 MHz, constructed a support vector regression prediction model based on soluble solid content, and the correlation coefficient of the model prediction reached 0.883^27^. The above studies have fully confirmed the feasibility and reliability of the dielectric property method in fields such as agricultural product quality detection and compost parameter monitoring. However, to date, there have been no research reports on dedicated dielectric sensors for the detection of typical veterinary drugs in paddy field water, leaving an obvious research gap in the relevant technology. Therefore, more in-depth exploration and research were needed for the detection methods of typical veterinary drug content in paddy field water, which will facilitate the realization of online monitoring of veterinary drug content in water bodies. This study aimed to design an artificial neural network-based sensor for veterinary drug detection in paddy field water and establish associated prediction models. The sensor used the STM32F405RGT6 chip as the core controller, with the main hardware circuit consisting of an excitation signal module, detection module, signal processing module, input–output module, boost-buck module, lithium battery charging module and interdigitated electrodes. It employed interdigitated electrodes as the detection probe and is powered by a lithium battery and solar panel. The sensor’s detection results could be wirelessly transmitted to a host computer via a LoRa coupled with 4G module. The content of veterinary drugs in paddy water could be directly observed on the host computer page, enabling real-time monitoring of paddy water bodies.

## Design of a sensor for detecting typical veterinary drugs

### Overall design of the veterinary drug detection sensor for paddy field water

**Fig. 1:**
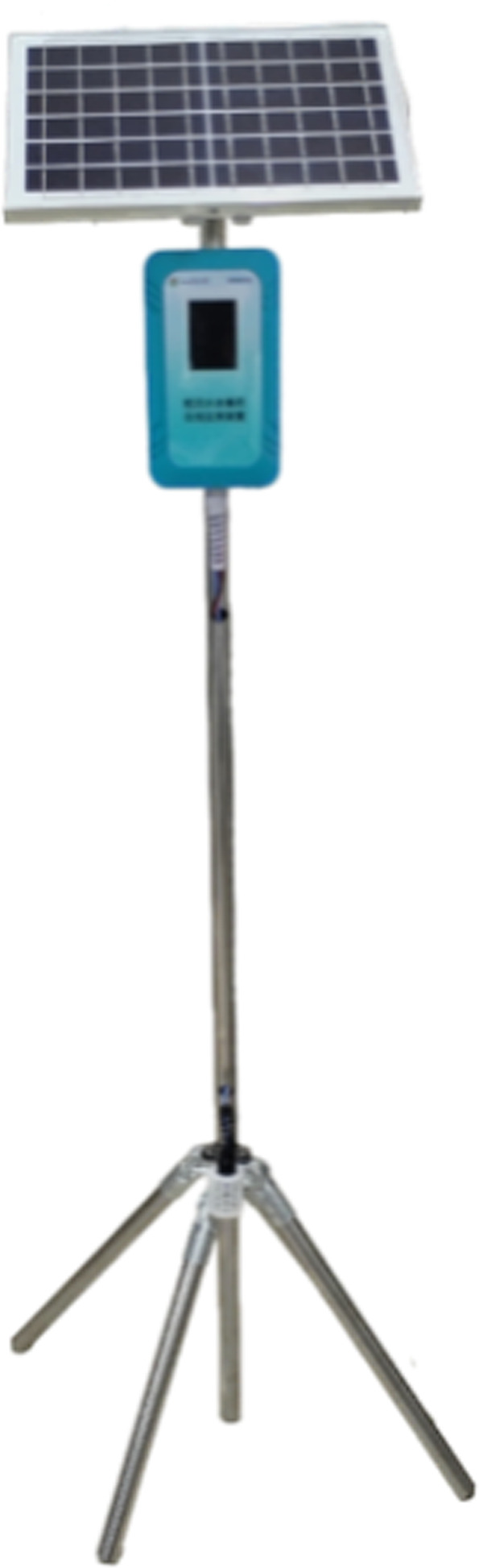
Veterinary drug detection sensor for paddy field water.

The veterinary drug detection sensor (Fig. 1) for paddy field water was primarily composed of the following components: solar panel, instrument box, main pole, four-leg support frame, interdigitated electrodes and waterproof connectors. The main pole and four-leg support frame were made of stainless steel. The four stainless steel tubes were fixed around the main pole using connectors, and the overall height of the sensor could be slightly adjusted by changing the angle between the stainless steel tubes and the main pole. Additionally, all connecting parts were selected for their excellent waterproof performance to ensure the reliable operation of the entire sensor in humid environments.

### Working principle of the sensor based on dielectric properties

Veterinary drug molecules in paddy field water, which contain different polar functional groups, altered the degree of water polarization and charge transfer characteristics after dissolution, thereby leading to changes in the water’s dielectric constant (ε); furthermore, changes in the type or content of veterinary drugs further induced corresponding variations in the dielectric properties of paddy field water. Based on this characteristic, this study adopted interdigital electrodes as detection probes and applied equal-amplitude sine wave signals ranging from 200 Hz to 100 MHz through an excitation signal module. When the electric field acted on the paddy field water containing veterinary drugs, the polar molecules in the water underwent oriented arrangement along the direction of the electric field, while the ions underwent directional migration. This process caused changes in the amplitude and phase of the output signals between the electrodes, which were ultimately converted into quantifiable amplitude ratio and phase difference parameters.

Due to the inherent differences in molecular polarity, the number of functional groups, and spatial configuration among different veterinary drugs, their regulatory amplitudes on the dielectric constant (ε) of paddy field water varied, which manifested as significant specificity in the amplitude ratio and phase difference signals at characteristic frequency points. By leveraging an artificial neural network (ANN) to learn the nonlinear mapping relationships among “signal characteristics—veterinary drug types—concentrations”, the simultaneous identification and quantitative detection of four target veterinary drugs were achieved.

### Main control hardware circuit design

**Fig. 2:**
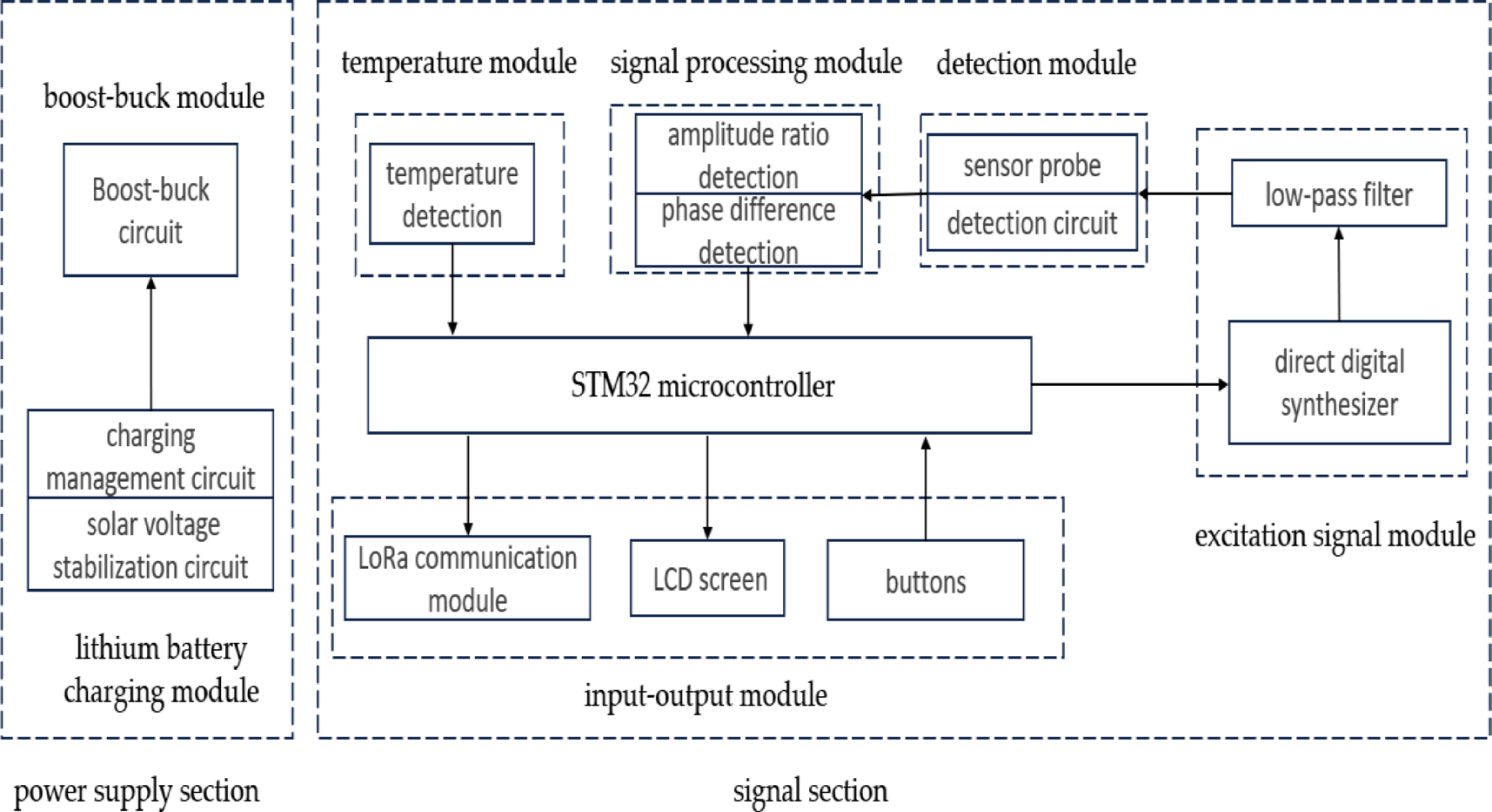
Overall design diagram of hardware circuit.

The hardware circuit (Fig. 2) of this sensor was divided into a power supply section and a signal section. The STM32 microcontroller, excitation signal module, detection module, and signal processing module in the signal section were the most critical components of the entire sensor and were essential for detecting veterinary drugs in paddy field water. The STM32 microcontroller primarily ensured that the sensor can properly collect and process data, as well as perform algorithm control. This sensor used the STM32F405RGT6 chip as the core controller. The excitation signal module generated excitation signals under the control of the STM32 microcontroller, producing stable sine wave signals within the range of 200 Hz to 120 MHz. The detection module employed interdigitated electrodes as the detection probe to collect data. The signal processing module converted amplitude ratio voltage and phase difference voltage into amplitude ratio and phase difference. The temperature module utilized the DS18B20 digital temperature sensor to detect water temperature. The input–output module included LoRa wireless transmission, LCD display and buttons. The power supply section consisted of a boost-buck module and a lithium battery charging module. The sensor was powered by a lithium battery with a voltage of 4.2 V. Since some modules in the circuit required voltages of 5 V, 3.3 V and 1.8 V, a boost module was used to increase the battery voltage from 4.2 V to 5 V and a buck module was used to step down the 5 V voltage to 3.3 V and 1.8 V.

### Interdigitated electrodes

**Fig. 3:**
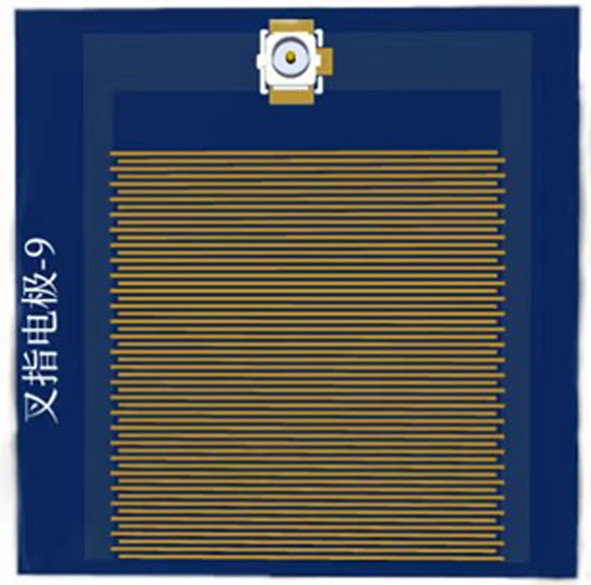
Interdigitated electrode.

Interdigitated electrodes (Fig. 3) were constructed on a resin substrate using conductive materials such as gold and copper to form multiple pairs of electrodes. The electrodes feature an interlocking finger-like design, with one serving as the excitation electrode to supply voltage and the other as the reference electrode. When a voltage signal of a certain amplitude is applied to the excitation electrode, the electric field causes ions in the solution to move, generating a current between the electrodes^28^.

### Analysis of main influencing factors of interdigitated electrodes

The capacitance of an interdigital electrode is mainly composed of two parts: fringing capacitance and parallel plate capacitance. Among them, the fringing capacitance is dominant and serves as the primary source of the total capacitance of the interdigital electrode. The classical approximate formula for fringing capacitance is as follows:

1 $$C = 2\left( {N~ - 1} \right)\varepsilon L \cdot ln\left( {1 + \frac{{2w}}{s} + \sqrt {\left( {\frac{{2w}}{s}} \right)^{2} + \frac{{4w}}{s}} } \right)$$

where: C: Capacitance value, N−1: Number of electrode pairs, ℇ: dielectric constant, L: Effective overlapping length of interdigital electrodes, S: Gap width between two adjacent interdigital electrodes, W: Width of a single interdigital finger.

It can be concluded from the above formula that with the plate area fixed, the smaller the electrode spacing, the larger the capacitance. In addition, the smaller the electrode width, the larger the capacitance.To ensure the stability of the detection signal from the interdigitated electrodes and further improve the accuracy of the detection system, the main influencing factors during the detection process need to be analyzed and discussed. Through comparative analysis, the optimal conditions for detection using interdigitated electrodes were determined. The two primary influencing factors for the interdigitated electrodes were the electrode width and the spacing between adjacent electrodes.

#### Influence of electrode spacing on frequency response

**Fig. 4:**
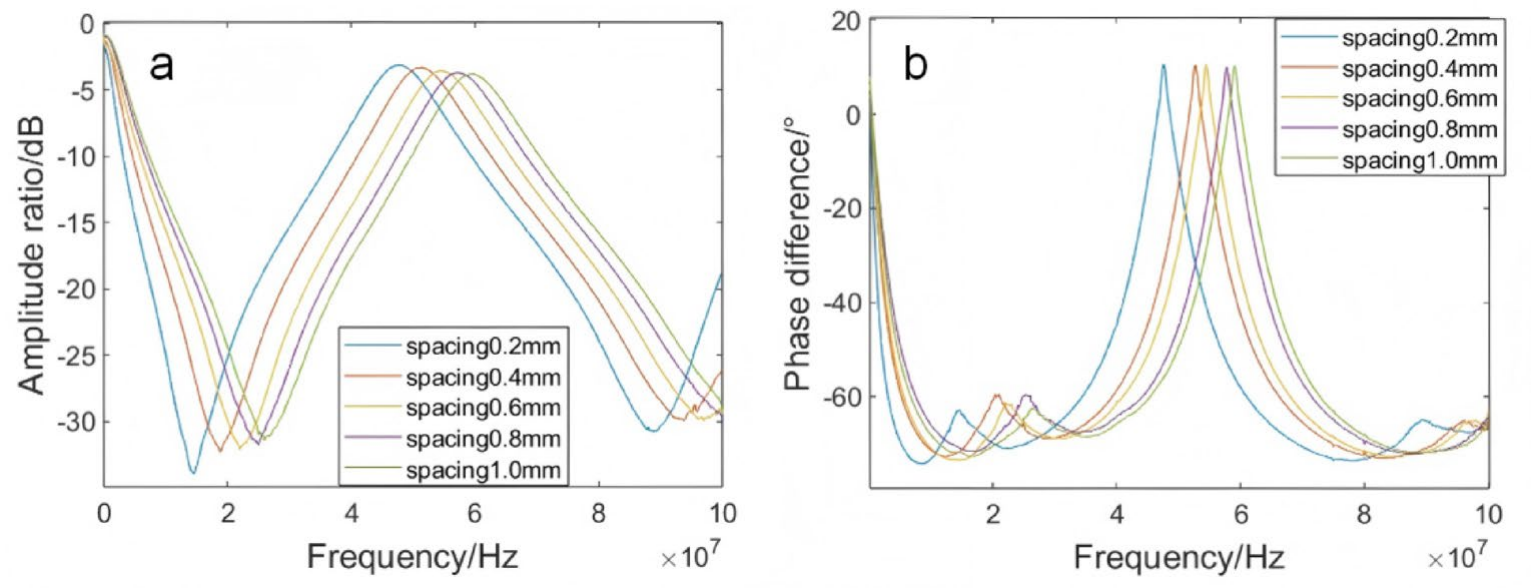
Frequency response curves of ultra-pure water at different electrode spacings. (**a**) amplitude ratio frequency response curve; (**b**) phase frequency response curve.

The spacing between electrodes was one of the critical factors affecting the capacitance of interdigitated electrodes. When the plate area was fixed, the smaller the electrode spacing, the greater the capacitance. To determine the optimal spacing for interdigitated electrodes, a 50 mL ultra-pure water solution containing 20 mg/L of ofloxacin was prepared. While keeping all other influencing factors constant, only the electrode spacing of the interdigitated electrodes was varied. Under the condition that all other influencing factors remain the same, only the electrode spacing of the interdigitated electrodes was altered. The frequency response curves of interdigitated electrodes with spacings of 0.2 mm, 0.4 mm, 0.6 mm, 0.8 mm and 1 mm were obtained by detecting the ofloxacin-containing ultrapure water solution, as illustrated in Fig. 4. In the amplitude ratio frequency response curve (Fig. 4a) within the 10 ~ 30 MHz frequency range, the amplitude ratio exhibited a negative correlation with the spacing, decreasing as the spacing increased. The strongest attenuation peak was observed at a spacing of 0.2 mm. Additionally, it was found that as the spacing increased, the peak positions of the frequency shifts gradually moved to the right. In the phase difference frequency response curve (Fig. 4b) within the 7 ~ 20 MHz frequency range at the first attenuation peak, the peak positions also shifted to the right as the spacing increased and the peak values decreased accordingly. Based on a comprehensive analysis of the detection results, the experimental performance was optimal when the electrode spacing was 0.2 mm. Therefore, the electrode spacing was ultimately determined to be 0.2 mm.

#### Influence of electrode width on frequency response

**Fig. 5:**
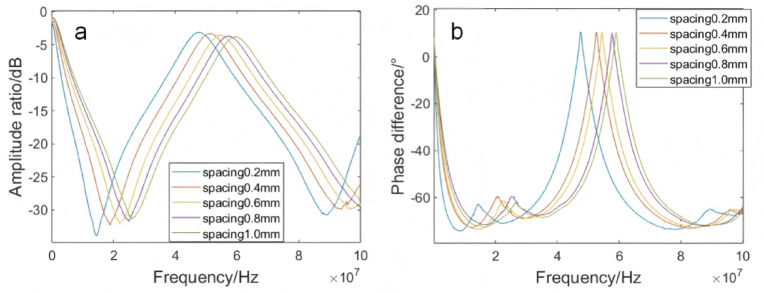
Frequency response curves of ultra-pure water at different electrode widths. (**a**) amplitude ratio frequency response curve; (**b**) phase frequency response curve.

The width of the electrodes was one of the critical factors influencing the capacitance of interdigitated electrodes. When the plate area was fixed, the smaller the electrode width, the greater the capacitance. To determine the optimal width for interdigitated electrodes, a 50 mL ultra-pure water solution containing 20 mg/L of ofloxacin was prepared. While keeping all other influencing factors constant, only the electrode width of the interdigitated electrodes was varied. Under the condition that all other influencing factors remained the same, only the electrode width of the interdigitated electrodes was altered. The frequency response curves of interdigitated electrodes with widths of 0.2 mm, 0.4 mm, 0.6 mm, 0.8 mm and 1 mm were obtained by detecting the ofloxacin-containing ultrapure water solution, as illustrated in Fig. 5. Upon observation of the amplitude ratio frequency response curves, it was noted that within the 10 ~ 20 MHz frequency range of the amplitude ratio frequency response curve (Fig. 5a), the amplitude ratio was observed to decrease negatively correlated with the increase in width, and the peak position was seen to gradually shift to the right. In the phase difference frequency response curve (Fig. 5b) within the 7 ~ 13 MHz frequency range at the first attenuation peak, the peak positions also shifted to the right as the width increased and the peak values decreased accordingly. Based on a comprehensive analysis of the detection results, the experimental performance was optimal when the electrode width was 0.2 mm. Therefore, the electrode width was ultimately determined to be 0.2 mm.

### External filtration device

**Fig. 6:**
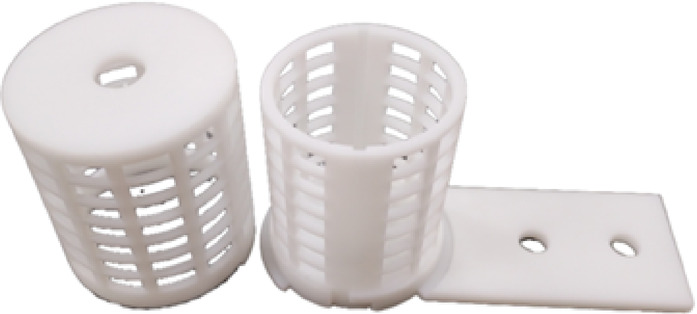
Filtration device.

The filtration device (Fig. 6) features both outer and inner layers designed in a cylindrical shape, secured together via snap-fit connections. The inner layer had an L-shaped groove and the outer layer had a protruding block whose width matches the L-shaped groove, ensuring a snug fit that prevents the outer layer from wobbling or detaching. Both the outer and inner layers of the filtration device were designed with a mesh structure to increase the filtration area. Filter cotton was placed between the outer and inner layers. The interdigitated electrodes were housed inside the inner layer, which had two grooves designed to the exact width and height to securely hold the electrodes in place, minimizing movement. The bottom of the inner layer extended outward, featuring two holes that allowed the filtration device to be screwed onto a support bracket. The entire device was designed to be straightforward and easy to understand.

### Application design for predicting typical veterinary drug concentrations in paddy field water

The predictive model was ported to the STM32F405RGT6. After lightweight optimization, its size was reduced to less than 100 KB and it could be directly called during program execution. The running time for a single set of veterinary drug concentration prediction was less than 1 min. Combined with the optimized characteristic frequency point detection strategy, the total duration of a single sensor detection was controlled within 4–6 min, which was significantly superior to that of traditional detection methods such as liquid chromatography-mass spectrometry (10–25 min) and immunoassay (15–30 min). This fully met the timeliness requirements for on-site real-time monitoring in paddy fields.The application phase program primarily consisted of the following components: initialization program, LoRa configuration program, AD9859 frequency sweep program, AD conversion program, serial port transmission program, temperature detection program, model invocation program and LCD display program.

**Fig. 7:**
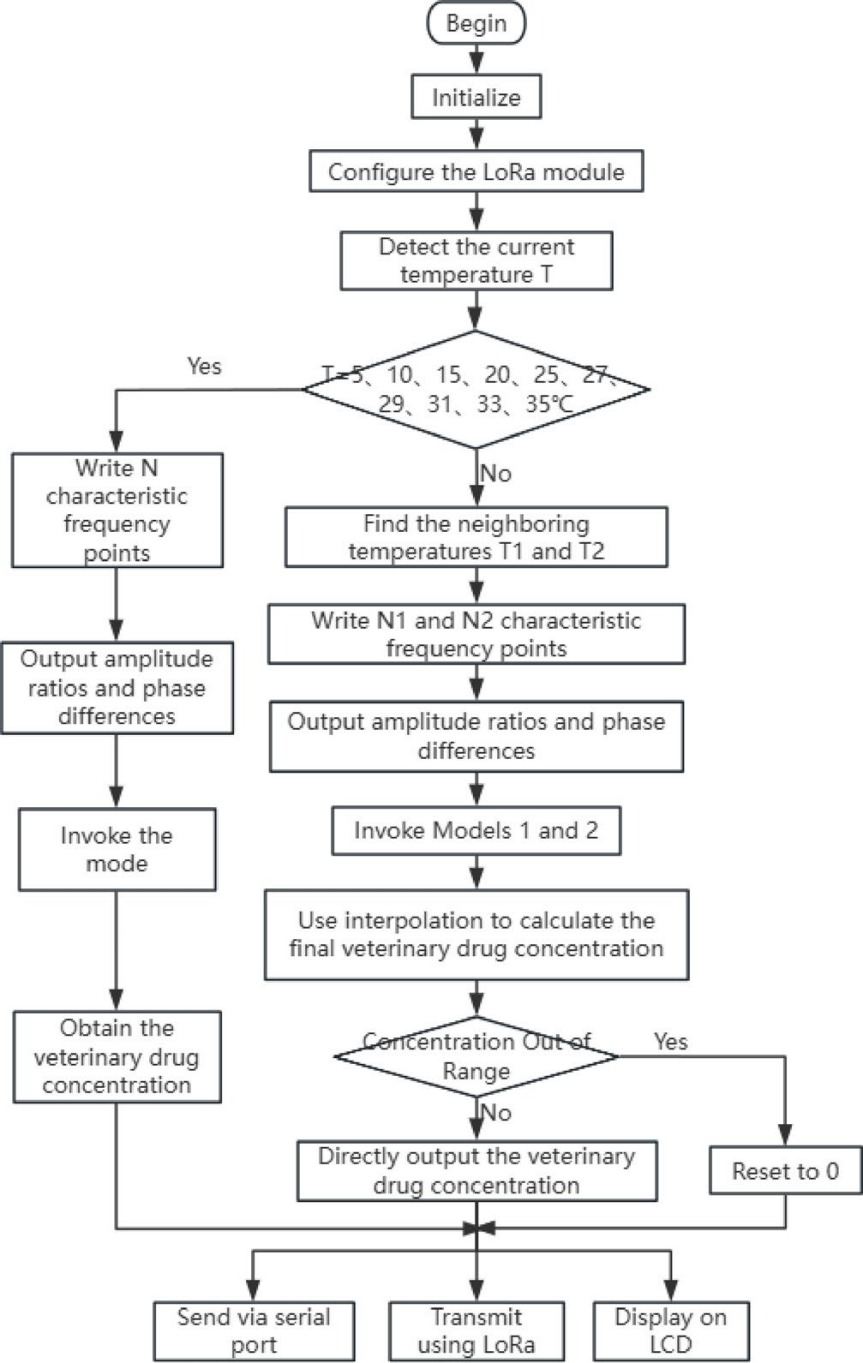
The flowchart of the application phase program.

In the program flowchart (Fig. 7), the initialization program was used to configure the peripherals and GPIO ports utilized by the STM32 chip. The LoRa configuration program was responsible for setting the working mode of the LoRa module, including the working channel, transmission power and related networking information. The temperature detection program acquired the current water temperature using the DS18B20 digital temperature sensor. The model invocation program first determined whether the detected current water temperature T matches one of the ten predefined temperatures: 5 °C, 10 °C, 15 °C, 20 °C, 25 °C, 27 °C, 29 °C, 31 °C, 33 °C, or 35 °C. If a match was found, the corresponding model for that temperature was invoked. If no match was found, the two closest temperatures were identified—one slightly higher T_1_ and one slightly lower T_2_ than the current temperature. The models corresponding to T_1_ and T_2_ were then invoked to obtain two sets of veterinary drug concentrations. These concentrations were substituted into an interpolation formula to calculate the final veterinary drug concentration. For example, for tetracycline hydrochloride, the two concentrations C_1_ and C_2_ obtained from the models are substituted into the interpolation formula to calculate the final concentration (C)^29^. The interpolation formula was as follows:

2 $$C = C_{2} + \frac{{T - T_{2} }}{{T_{1} - T_{2} }}\left( {C_{1} - C_{2} } \right)$$

where: T: Detected current temperature. T_1_: Predefined temperature slightly higher than T. T_2_: Predefined temperature slightly lower than T. C: Veterinary drug concentration at the current temperature. C_1_: Veterinary drug concentration obtained from model 1. C_2_: Veterinary drug concentration obtained from model 2.

It then checked whether the predicted veterinary drug concentration exceeds the defined concentration range. If it did, the concentration was set to zero. The LCD display program showed the temperature and prediction resulted in real-time on the LCD screen. In practical use, the debugged paddy field water veterinary drug monitoring sensor was deployed in a paddy field environment. Turn on the switch, waited for the sensor to detect and when the detection result was obtained, the veterinary drug concentration would be displayed on the LCD screen of the sensor. Simultaneously, the concentration was uploaded to the host computer via the LoRa coupled with 4G module, allowing real-time monitoring of the detected veterinary drug concentration in the paddy field water on the host computer.

#### LoRa configuration program

The LoRa configuration program is primarily designed to set critical operational parameters of the LoRa module, including baud rate, transmission rate, operating mode, and communication channel. The module features four distinct working modes selected through logic level combinations of the M0 and M1 control pins: transmission mode (M0 = 0, M1 = 0) enables both serial communication and wireless transmission functions; WOR mode (M0 = 1, M1 = 0) allows definition of transmitter and receiver roles; configuration mode (M0 = 0, M1 = 1) permits register access via serial interface for parameter adjustment; while sleep mode (M0 = 1, M1 = 1) minimizes power consumption. In the current implementation, only transmission mode and configuration mode were employed. The configuration procedure requires initial entry into configuration mode to establish uniform parameters across communicating modules, ensuring synchronization of baud rate (9600 bps), parity mode (8N1), network address (1), over-the-air rate (62.5 kbps), and channel frequency (channel 52 in transparent transmission mode). This parameter alignment is essential for establishing successful wireless communication links between modules during operational transmission mode. The standardized configuration adopted in this study guarantees reliable data exchange while maintaining system compatibility and performance consistency.

#### Sweep program

**Fig. 8:**
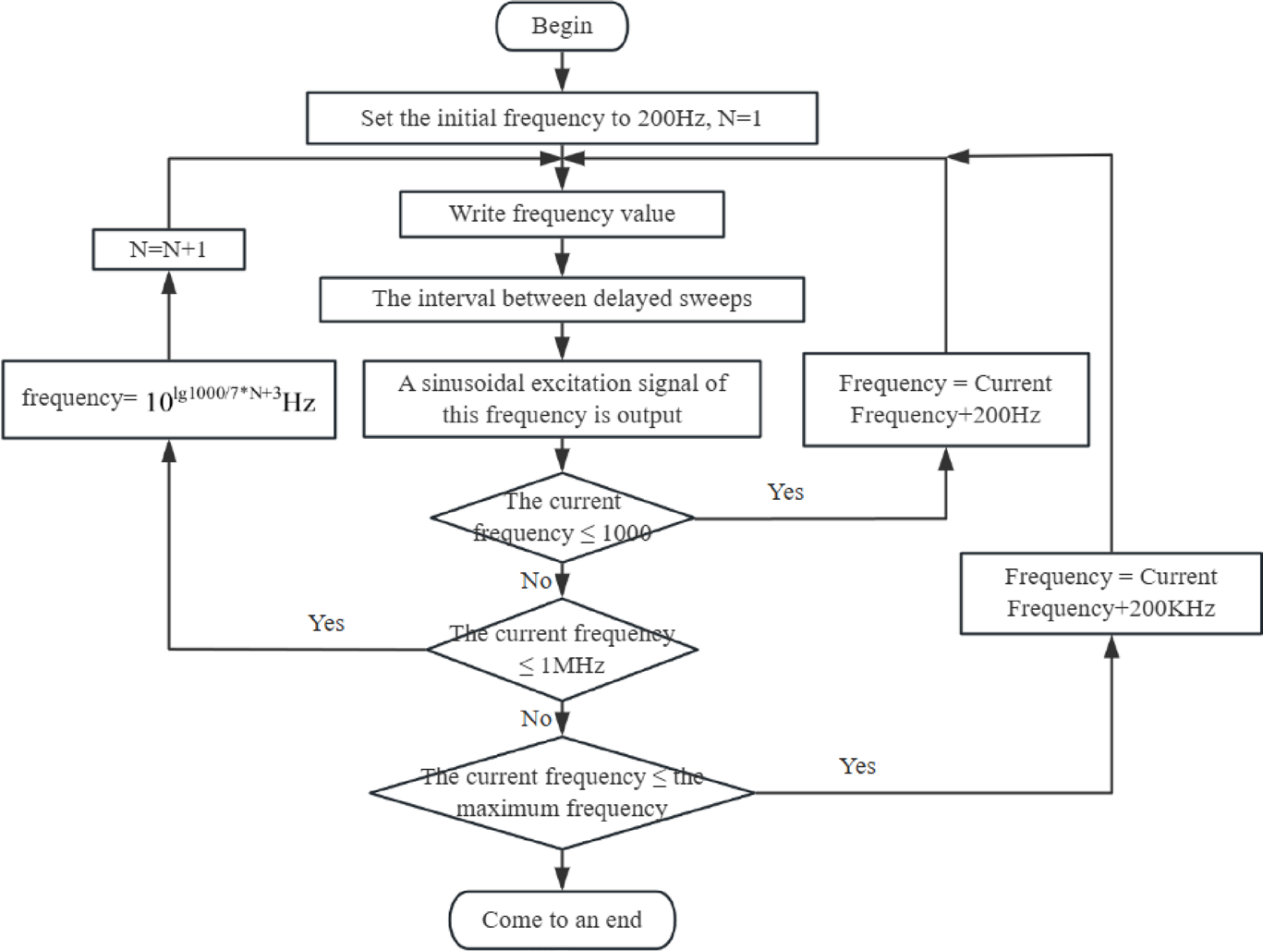
Sweep procedure during the data acquisition phase.

The core principle of detecting paddy field water using interdigital electrodes lies in analyzing the frequency response characteristics between the electrodes, which requires an excitation signal as the reference benchmark. By observing the changes in amplitude ratio, phase difference, and their combined effects when a sinusoidal signal passes through the paddy water, the detection is achieved. To support multi-frequency point measurements, this study employs digital frequency scanning technology, enabling the signal source to adjust its output frequency according to programmed configuration parameters. The flowchart of the frequency scanning program in the data acquisition phase was shown in Fig. 8. The AD9859 direct digital synthesis (DDS) chip is selected as the signal generator, which can output sine signals up to 200 MHz. To ensure signal quality, the maximum output frequency of the AD9859 chip was set at 100 MHz in this study. During the data acquisition phase, a three-segment frequency sweep strategy was employed. The low-frequency range (200 Hz–1 kHz) was scanned from an initial frequency of 200 Hz with a step increment of 200 Hz, collecting 5 frequency points. The mid-frequency range (1 kHz–1 MHz) was sampled at 7 logarithmically equidistant points. The high-frequency range (1 MHz–100 MHz) was scanned with a step size of 200 kHz, acquiring 495 frequency points. During the application phase, the frequency sweep procedure was optimized to perform detection only at characteristic frequency points, thereby significantly reducing the scanning time. As a result, the sensor achieved rapid detection of veterinary drug residues in paddy water within 4–6 min per test, while liquid chromatography-mass spectrometry (LC–MS) requires 10–25 min, and immunoassay methods take 15–30 min for a single detection. Compared to other detection methods, this sensor is significantly faster.

#### AD Conversion Progrm

Since the main control chip cannot directly process analog signals, an ADC module is required to convert the analog output from the AD8302 gain-phase detector into digital signals, thereby obtaining the amplitude-frequency and phase-frequency characteristics between the detection probe and the excitation signal. The on-chip ADC1 channels 10 and 11 of the STM32F405RGT6 microcontroller were utilized to collect the frequency response characteristics of paddy water. The signal acquisition process involves configuring a storage buffer, performing continuous sampling, and storing the collected data in an array. Upon reaching the preset sampling volume, the effective data is filtered through a bubble-sorting algorithm to remove the maximum and minimum values, followed by calculating the arithmetic mean of the standardized dataset as the final measurement value. After completing the AD conversion, the acquired voltage values are further converted into corresponding amplitude ratio and phase difference parameters based on the conversion relationship specified in the AD8302 technical manual. The specific conversion formulas are as follows:

3 $$AMP = - \left[ {\frac{{ADC1_{ - } 10}}{{2^{12} }} \times \frac{3300}{{30}}} \right] + 30\left( {db} \right),$$

4 $$PHS = - \left[ {\frac{{ADC1_{ - } 11}}{{2^{12} }} \times \frac{3300}{{30}}} \right] - 180\left(^\circ \right),$$

In the formulas: AMP: Amplitude ratio value; ADC1_10: Conversion value of on-chip ADC1 channel 10; PHS: Phase difference value; ADC1_11: Conversion value of on-chip ADC1 channel 11.

#### Model calling program

The prediction model based on artificial neural network employs a hierarchical architecture, where each layer’s nodes receive excitation signals from preceding layers and propagate the weighted processed signals to subsequent layers. Through continuous parameter optimization via backpropagation algorithm, the model progressively approximates the functional relationship between inputs and outputs. After adequate training, the model demonstrates excellent generalization capability for effective prediction on unseen data. In the implementation, the Run function executes the model using acquired data as input (pIn) and outputs the concentrations of four veterinary drugs (pOut), with model selection determined by real-time paddy water temperature monitoring. The term ‘network’ specifically denotes the 5 °C veterinary drug concentration prediction model deployed on the code for model invocation is as follows:


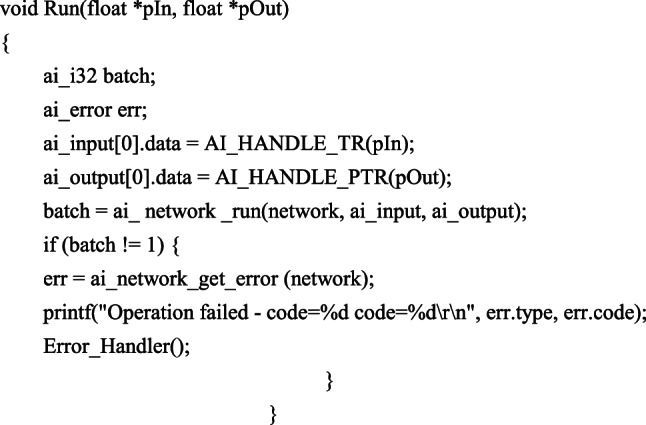


### Toxicological risks and environmental impacts

This study also conducted a systematic evaluation of the toxicological risks and environmental impacts associated with the operation of the sensor in paddy field environments. In terms of toxicological risks, the potential hazards during the sensor’s operation stemmed primarily from exposure to target analytes rather than the sensor’s own components. Through hardware encapsulation design, operational protection protocols (such as wearing gloves, masks, and other protective equipment), and standardized waste liquid treatment (neutralization and dilution to meet discharge standards), the risks of human irritation and ecological accumulation caused by veterinary drugs via skin contact, inhalation, or environmental leakage were effectively mitigated.Regarding environmental impacts, the sensor adopted a dual power supply mode (lithium battery + solar panel), enabling continuous operation for 7–10 days on a single charge with no greenhouse gas emissions. Its main structure was fabricated using environmentally friendly materials such as stainless steel and degradable resin, while metal components were recyclable and would not cause soil or water pollution after disposal. During deployment, the sensor only needed to be inserted into the topsoil layer (≤ 20 cm), generating no noise and requiring no addition of chemical reagents. It exerted no significant interference on rice growth, aquatic organisms, or microbial communities in paddy fields.

In summary, the sensor met the demand for rapid detection while exhibiting excellent environmental compatibility and safety, which provided crucial support for its large-scale field application.

## Materials and methods

### Experimental materials and equipment

Experimental materials were as following in this paper: Ultrapure water, paddy water was collected from the experimental fields of Jiangxi Agricultural University on July 27, 2023, with a maximum temperature of 35 °C and a minimum temperature of 26 °C. The four veterinary drugs, i.e., sulfamerazine (SMR), doxycycline hydrochloride (DCH), ofloxacin (OFL) and tetracycline hydrochloride (TC), were all of analytical standard grade. Sulfamerazine was purchased from Shanghai Yuanye Bio-Technology Co., Ltd., with HPLC purity ≥ 98% and CAS number 127-79-7. Doxycycline hydrochloride was purchased from Aladdin, with HPLC purity ≥ 98% and CAS number 24390-14-5. Ofloxacin was purchased from the National Standards Material Network, with HPLC purity ≥ 99% and CAS number 82419-36-1. Tetracycline hydrochloride was purchased from Beijing Solarbio Science & Technology Co., Ltd., with HPLC purity ≥ 99% and CAS number 64-75-5.

Instrumentation and equipment were as following in this paper: Paddy water veterinary drug detection sensor, interdigitated electrodes (size 30 mm × 30 mm), 20 mL brown volumetric flask, 25 mL brown volumetric flask, 50 mL brown volumetric flask, 100 mL brown volumetric flask, 200 mL brown volumetric flask, cylindrical lidless acrylic tube (outer diameter 40 mm, height 100 mm, thickness 2 mm), 0.3 mm thick filter cotton, low-temperature constant temperature water bath (DC-0506, Jiangsu Tianling Instrument Co., Ltd.), Kylin-Bell vortex mixer (VORTEX-5, Qilin Bell Instrument Manufacturing Co., Ltd.), ultrapure water system (Teledyne Tekmar, USA), glass stirring rod, container cleaning brush, volumetric flask brush, test tube rack, ultrasonic cleaner (JK-50B, Hefei Jinike Mechanical Manufacturing Co., Ltd.), electronic balance (FA1004B, Beijing Kewei Yongxing Instrument Co., Ltd.), high-precision pipettes (Thermo Fisher Scientific; Dragon Lab Instruments (Beijing) Co., Ltd.) and lithium battery-powered water pump (Shanghai Xiannan Industrial Co., Ltd.).

### Overview of test samples for paddy field water

Temperature was regarded as one of the important factors that affect the dielectric properties of a medium. The minimum temperature for rice seedling germination was 10 to 12 °C; The suitable water temperature during the early growth stage was 22 to 28 °C; The optimal growth temperature was 28 to 32 °C; The suitable water temperature during the mid-growth stage was 28 to 32 °C and the optimal temperature for heading was 25 to 35 °C. Therefore, ten temperature levels (i.e., 5 °C, 10 °C, 15 °C, 20 °C, 25 °C, 27 °C, 29 °C, 31 °C, 33 °C and 35 °C) were set to detect typical veterinary drugs in paddy field water.

#### Overview of test samples for paddy field water containing single veterinary drug

The single veterinary drug experiment focused on the types and concentrations of four veterinary drugs, investigating the changes in frequency response characteristics when paddy field water contained only one of these veterinary drugs. For each veterinary drug, samples with 10 different temperatures and varying concentrations were tested. The concentration range for sulfamerazine samples was established from 1 to 90 mg/L, for doxycycline hydrochloride samples from 1 to 120 mg/L, for ofloxacin samples from 1 to 120 mg/L, and for tetracycline hydrochloride samples from 2 to 240 mg/L. For each veterinary drug, 100 samples were prepared at each single temperature and a total of 1000 samples were obtained across 10 temperatures. Therefore, the total number of samples for all four single veterinary drugs combined was 4000.

#### Overview of test samples for paddy field water containing mixed veterinary drug

The mixed veterinary drug experiment was conducted to test random combinations of four veterinary drugs. The paddy water solution with multiple veterinary drugs added was analyzed to explore whether the content of each drug in the mixed solution could be detected. The mixing methods of the four veterinary drugs included two-drug mixtures, three-drug mixtures and four-drug mixtures. There were 11 mixing methods: 6 for two-drug mixtures, 4 for three-drug mixtures and 1 for four-drug mixtures. For different mixing methods of the drugs, mixtures with different concentrations and at different temperatures were required. The different concentrations included three gradients: low, medium and high. In the two-drug mixture experiments, there were 6 mixing methods. For each mixed veterinary drug at a single temperature, 40 samples were prepared and a total of 2400 samples were accumulated for the 6 mixed drugs across 10 temperatures. In the three-drug mixture experiments, there were 4 mixing methods. For each mixed veterinary drug at a single temperature, 40 samples were prepared and a total of 1600 samples were accumulated for the 4 mixed drugs across 10 temperatures. In the four-drug mixture experiment, there was only 1 mixing method. For each mixed veterinary drug at a single temperature, 90 samples were prepared and a total of 900 samples were accumulated for this mixed drug across 10 temperatures. In summary, the total number of samples for the mixed veterinary drug experiments was 4900.

### Frequency response data acquisition

The samples were placed in a constant temperature water bath to ensure that the paddy field water samples were always maintained at the set temperature. During the detection, all interdigital electrodes were immersed in the water samples. The acquisition frequency was set to 200 Hz ~ 100 MHz, with 507 frequency points scanned and a sampling interval of 100 ms.

### Data processing

#### Feature extraction method

In the paddy field water veterinary drug detection experiment, 507 sets of raw data were obtained each time the detection of a paddy field water sample containing veterinary drugs was completed. The experiment took different temperatures as variables and conducted systematic detection on paddy field water samples containing different types and concentrations of veterinary drugs, thus generating a large-scale dataset. Since massive data might contain invalid information, which would easily interfere with the accuracy of results if directly used for analysis, it was necessary to screen the data first, eliminate the invalid information and retain the valid experimental data. Based on this, this study adopted the competitive adaptive reweighted sampling (CARS) feature extraction method to accurately extract effective frequency points from the raw data, laying a foundation for subsequent data analysis.

CARS was a method that imitated the evolutionary principle of survival of the fittest in nature to establish variable selection^30^. The specific process involved setting the number of sampling times N and conducting sampling in combination with the highly random Monte Carlo method^31^. Before CARS feature extraction, the raw data was divided into a training set and a prediction set with a ratio of 3:1. Three types of data, namely amplitude ratio, phase difference, and amplitude ratio combined with phase difference, were subjected to CARS feature extraction, and the data after feature extraction was used to build the model.

#### Modeling method

The prediction model for veterinary drug content in paddy field water was modeled using Artificial Neural Network (ANN). ANN could fully approximate any complex non-linear relationship. Before modeling, the data was first divided into a training set and a prediction set. The training set underwent CARS feature extraction, and the training set after feature extraction was used for modeling. Then, the prediction set was used for prediction to obtain prediction results, and the performance of the established model was judged based on the prediction results.

## Results and discussion

### Frequency response analysis of veterinary drugs with different concentrations in paddy field water

**Fig. 9:**
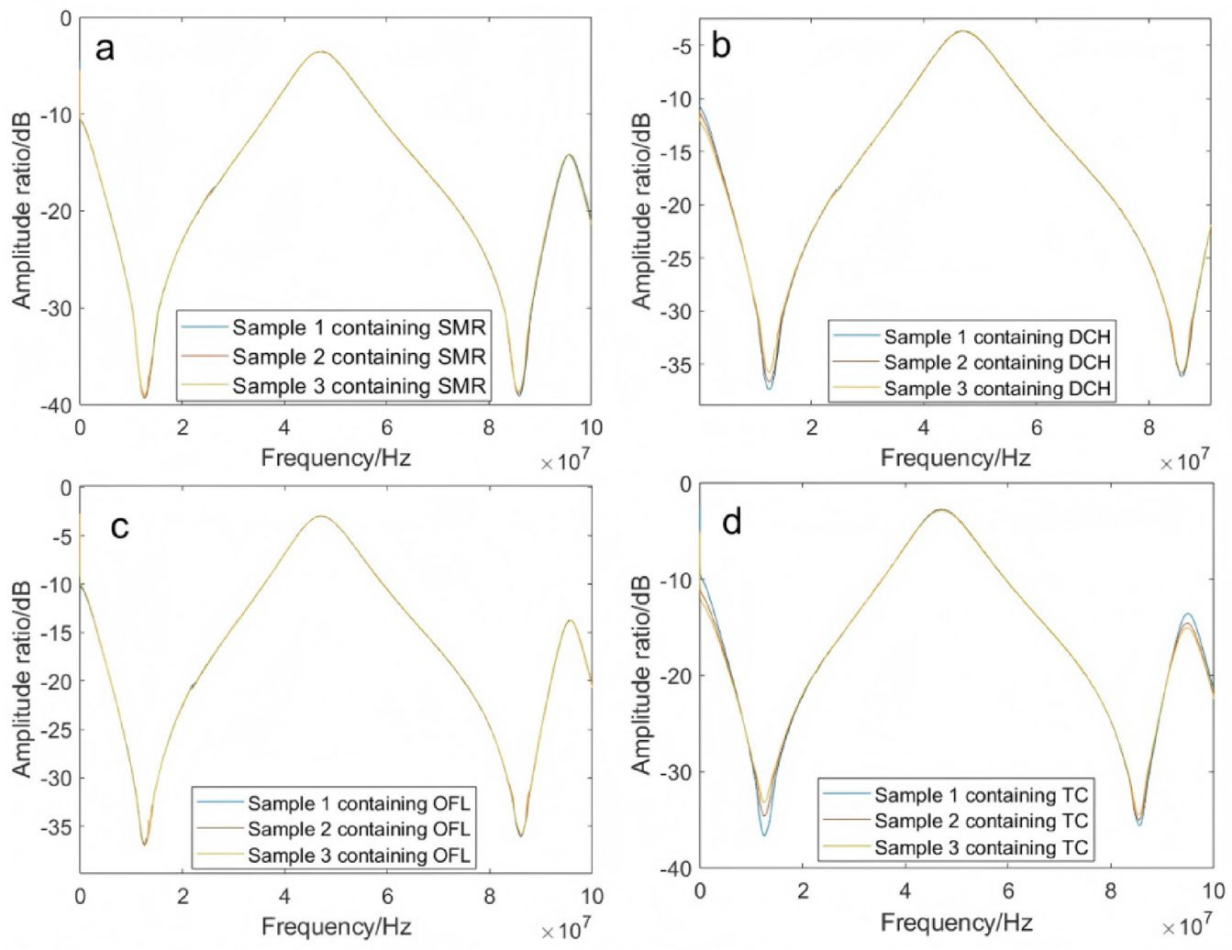
Frequency response curves of paddy water samples containing different concentrations of veterinary drugs (amplitude ratio). (**a**) Rice paddy water sample containing SMR; (**b**) Rice paddy water sample containing DCH; (**c**) Rice paddy water sample containing OFL; (**d**) Rice paddy water sample containing TC.

**Fig. 10:**
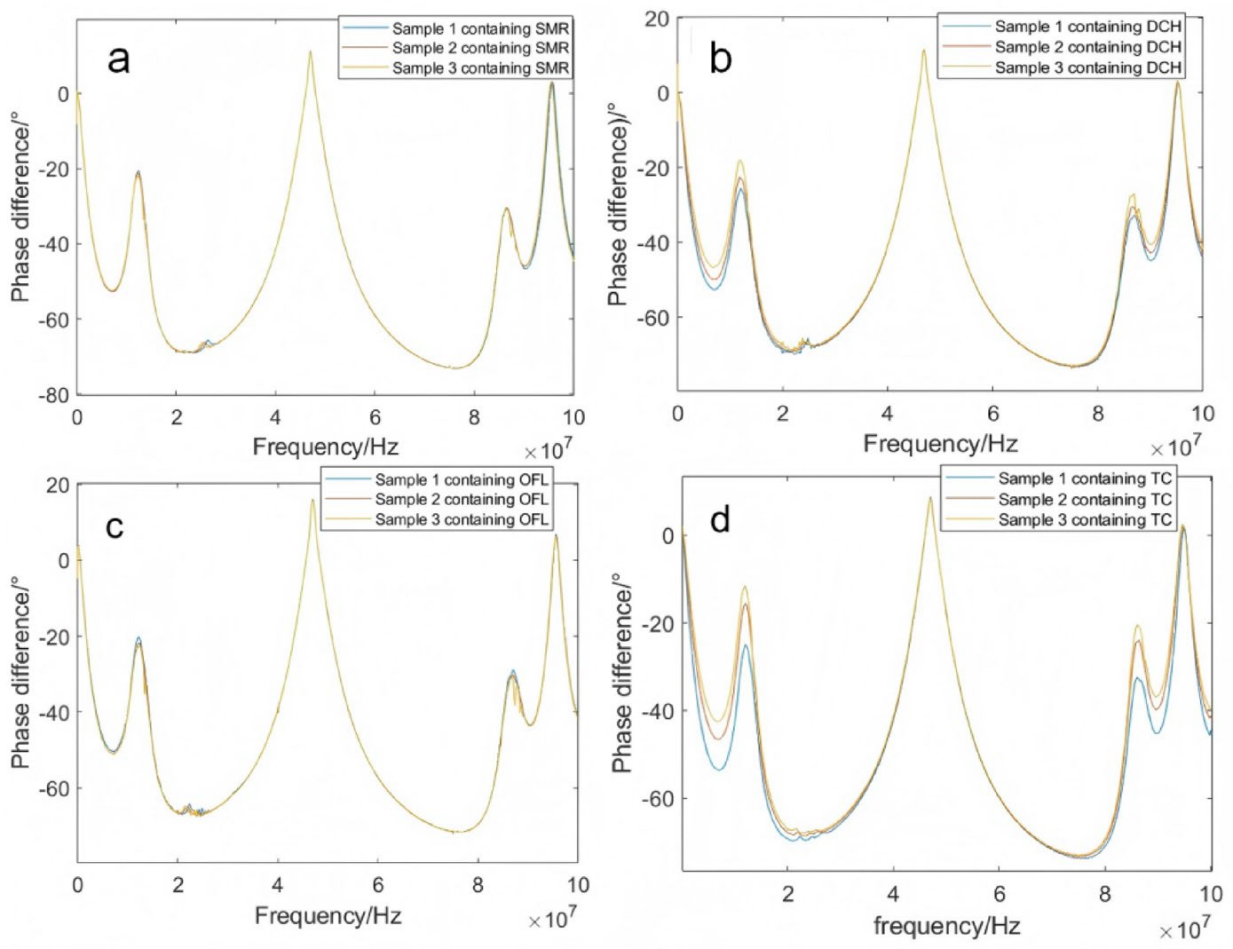
Frequency response curves of paddy water samples containing different concentrations of veterinary drugs (phase difference). (**a**) Rice paddy water sample containing SMR; (**b**) Rice paddy water sample containing DCH; (**c**) Rice paddy water sample containing OFL; (**d**) Rice paddy water sample containing TC.

This study conducted experiments on four veterinary drugs with 15 mixing methods at a temperature range of 0 to 35 °C, and data obtained at 5 °C was used as an example. Figures 9 and 10 showed the frequency response curves of paddy water samples containing sulfamethazine, doxycycline hydrochloride, ofloxacin, and tetracycline hydrochloride at different concentrations, measured at 5 °C.

By observing the amplitude-frequency response curves of paddy water samples containing different concentrations of sulfamethazine, doxycycline hydrochloride, ofloxacin, and tetracycline hydrochloride at 5 °C (Fig. 9), it was found that the amplitude-frequency response curves of these veterinary drug-containing paddy water samples exhibited significant changes in the frequency ranges of 200 Hz to 16 MHz and above 80 MHz. All amplitude attenuation peaks decreased, though the magnitude of the decrease varied with the type of veterinary drug.

In the phase-frequency response curves of paddy water samples containing different concentrations of sulfamethazine, doxycycline hydrochloride, ofloxacin, and tetracycline hydrochloride at 5 °C (Fig. 10), the phase-frequency response curves of these veterinary drug-containing paddy water samples (at varying concentrations) were presented. The effect of changes in veterinary drug concentration on the phase difference could be observed in the front and rear segments of the curves. The main changes occurred at the phase peaks: at 7 MHz and 89.6 MHz, paddy water samples containing doxycycline hydrochloride and tetracycline hydrochloride both exhibited attenuation peaks, with the peak values decreasing as the concentration of the veterinary drugs increased. The change in tetracycline hydrochloride was more pronounced than that in doxycycline hydrochloride. At 12 MHz and 86 MHz, paddy water samples containing sulfamethazine, doxycycline hydrochloride, ofloxacin, and tetracycline hydrochloride all showed enhancement peaks, with the peak values increasing as the concentration of the veterinary drugs increased. The magnitude of the increase varied with the type of veterinary drug, and tetracycline hydrochloride exhibited the most significant change.

### CARS feature extraction for the prediction of typical veterinary drug content in paddy water

In this study, the collected data included three types: amplitude ratio, phase difference, and the combination of amplitude ratio and phase difference. Predictive models for typical veterinary drug content in paddy water were established separately at 10 temperatures: 5 °C, 10 °C, 15 °C, 20 °C, 25 °C, 27 °C, 29 °C, 31 °C, 33 °C, and 35 °C. Moreover, for each veterinary drug content prediction model established at each temperature level, a multi-input multi-output (MIMO) architecture design was adopted to achieve efficient prediction of the target veterinary drug content.

The multi-input multi-output model could simultaneously output the concentrations of four veterinary drugs: sulfamethazine, doxycycline, ofloxacin, and tetracycline. Regarding the modeling data, a total of 892 sets of paddy water sample data were collected, including the amplitude ratio and phase difference data of blank paddy water samples at a specific temperature, as well as the amplitude ratio and phase difference data of 15 types of drug-containing paddy water samples prepared by mixing the above four veterinary drugs in 15 different ways. All data were divided into a training set and a prediction set at a ratio of 3:1, which were used for model training and performance verification. To optimize the modeling effect, feature extraction was conducted before model construction. Specifically, the Competitive Adaptive Reweighted Sampling (CARS) algorithm was applied in MATLAB R2023a software to perform feature selection on three types of feature data: amplitude ratio, phase difference, and amplitude ratio + phase difference. Table 1 below detailed the number of features extracted from the three types of data by the CARS algorithm. Each set of amplitude ratio/phase difference data contained 507 frequency points, which were reduced to 119–337 frequency points after CARS feature extraction.

**Table 1:** Numbers of CARS feature extraction under different temperature.

Temperature (℃)	5℃	10℃	15℃	20℃	25℃	27℃	29℃	31℃	33℃	35℃
Amplitude ratio	213	222	188	119	188	282	253	337	249	282
Phase difference	178	205	247	171	223	180	248	214	139	249
Amplitude ratio combined with phase difference	154	150	243	249	248	254	224	203	312	239

### Results and analysis of the prediction model for typical veterinary drug content in paddy water

In this study, 16 types of paddy water samples were tested using a detection device at 10 temperatures. Feature extraction was performed on three types of data: amplitude ratio, phase difference, and the combination of amplitude ratio and phase difference. A MIMO-based ANN prediction model for typical veterinary drug content in paddy water was established.

#### Prediction results and analysis of the multi-input multi-output model using amplitude ratio data

From Table 2, it could be observed that the concentration prediction model using amplitude ratio frequency response data as input performed best for sulfamethazine in paddy field water at 15 °C, with the highest prediction coefficient of determination (R^2^) of 0.6894. For doxycycline hydrochloride, the best prediction performance occurred at 5 °C, with the highest R^2^ of 0.7696. For ofloxacin, the best prediction performance was achieved at 5 °C, with the highest R^2^ of 0.728. For tetracycline hydrochloride, the best prediction performance was observed at 31 °C, with the highest R^2^ of 0.8843.

**Table 2:** Using the amplitude ratio as the input value to predict the performance of the model.

Temperature (℃)	Sulfamethazine		Ofloxacin		Doxycycline Hydrochloride		Tetracycline Hydrochloride	
	R^2^	RMSEP (mg/L)	R^2^	RMSEP (mg/L)	R^2^	RMSEP (mg/L)	R^2^	RMSEP (mg/L)
5	0.6263	21.8616	0.7696	23.2013	0.728	25.4490	0.8716	33.8290
10	0.6434	22.3005	0.631	29.2710	0.6747	28.6369	0.8183	39.8894
15	0.6894	20.2624	0.7185	25.9243	0.7267	24.9588	0.8718	33.5182
20	0.6674	20.8039	0.6822	28.2664	0.6933	26.1734	0.8297	40.0913
25	0.6772	21.5001	0.7302	25.0438	0.704	26.4712	0.8737	33.2758
27	0.6214	22.4920	0.6632	30.5344	0.6295	28.7754	0.8614	35.4914
29	0.5509	23.3501	0.6481	27.5759	0.5746	30.7594	0.8434	38.4007
31	0.6308	21.1240	0.6771	26.4835	0.5442	31.0085	0.8843	31.3390
33	0.559	24.2381	0.7411	24.5168	0.5323	34.8322	0.8795	33.8659
35	0.6753	19.6208	0.7077	25.1529	0.5884	29.5869	0.8157	39.5975

Overall, Table 2 showed that the prediction performance for tetracycline hydrochloride in paddy field water was the best, with the highest R^2^ of 0.8843 and the lowest R^2^ of 0.8157. The prediction performance for doxycycline hydrochloride ranked second, with the highest R^2^ of 0.7696 and the lowest R^2^ of 0.631. The prediction performance for ofloxacin was worse, with the highest R^2^ of 0.728 and the lowest R^2^ of 0.5323. The prediction performance for sulfamethazine was the poorest, with the highest R^2^ of 0.6894 and the lowest R^2^ of 0.5509. Table 2 presented all prediction results and analyses of the multi-input multi-output model for typical veterinary drugs in paddy field water—where the model used amplitude ratio data across 10 temperatures.

#### The prediction results and analysis of the multiple-input multiple-output model were established using phase difference data

From Table 3, it could be observed that the concentration prediction model using phase difference frequency response data as input performs best for sulfamethazine in paddy field water at 29 °C, with the highest prediction R^2^ of 0.7894. For doxycycline, the best prediction performance was at 33 °C, with the highest R^2^ of 0.8419. For ofloxacin, the best prediction performance was at 15 °C, with the highest R^2^ of 0.8027. For tetracycline hydrochloride, the best prediction performance was at 15 °C, with the highest R^2^ of 0.9205.

**Table 3:** Using the phase difference as the input value to predict the performance of the model.

Temperature (℃)	Sulfamethazine		Ofloxacin		Doxycycline Hydrochloride		Tetracycline Hydrochloride	
	R^2^	RMSEP (mg/L)	R^2^	RMSEP (mg/L)	R^2^	RMSEP (mg/L)	R^2^	RMSEP (mg/L)
5	0.7577	17.9281	0.826	20.1146	0.7169	25.8412	0.8978	30.1827
10	0.6683	21.8122	0.7684	23.7673	0.7743	22.7403	0.9027	29.4282
15	0.7493	18.4177	0.8279	19.9884	0.8027	21.4435	0.9205	27.4010
20	0.778	17.9579	0.8179	20.5780	0.7237	26.0276	0.8833	32.3691
25	0.7521	18.4440	0.7892	22.8079	0.731	24.5882	0.9065	29.8599
27	0.7088	20.9977	0.8048	21.3093	0.6422	29.3032	0.9183	27.7120
29	0.7894	16.5064	0.7469	24.2528	0.6469	28.7191	0.8569	35.6860
31	0.6793	20.8079	0.7856	22.3089	0.6435	28.4929	0.8952	31.7180
33	0.6443	21.3751	0.8419	19.8957	0.7395	24.9912	0.9114	29.0778
35	0.7014	21.5236	0.7552	23.9052	0.6443	28.7007	0.8516	36.2189

Overall Table 3 showed that the prediction performance for tetracycline hydrochloride in paddy field water was the best, with the highest R^2^ of 0.9205 and the lowest R^2^ of 0.8516. The prediction performance for doxycycline hydrochloride was the second best, with the highest R^2^ of 0.8419 and the lowest R^2^ of 0.7469. The prediction performance for ofloxacin was worse, with the highest R^2^ of 0.8027 and the lowest R^2^ of 0.6443. The prediction performance for sulfamethazine was the poorest, with the highest R^2^ of 0.7894 and the lowest R^2^ of 0.6443. Table 3 presented all prediction results and analyses of the multi-input multi-output model for typical veterinary drugs in paddy field water using phase difference data across 10 temperatures.

#### Prediction results and analysis of the multi-input multi-output model using amplitude ratio coupled with phase difference data

From Table 4, it could be observed that the concentration prediction model using both amplitude ratio and phase difference frequency response data as input performs best for sulfamethazine in paddy field water at 29 °C, with the highest prediction R^2^ of 0.6472. For doxycycline hydrochloride, the best prediction performance was at 25 °C, with the highest R^2^ of 0.69. For ofloxacin, the best prediction performance was at 15 °C, with the highest R^2^ of 0.6529. For tetracycline hydrochloride, the best prediction performance was at 31 °C, with the highest R^2^ of 0.8533.

**Table 4:** Using the amplitude ratio coupled with phase difference as the input value to predict the performance of the model.

Temperature (℃)	Sulfamethazine		Ofloxacin		Doxycycline Hydrochloride		Tetracycline Hydrochloride	
	R^2^	RMSEP (mg/L)	R^2^	RMSEP (mg/L)	R^2^	RMSEP (mg/L)	R^2^	RMSEP (mg/L)
5	0.5254	26.9878	0.5744	33.7233	0.5345	35.0309	0.7301	50.0621
10	0.6149	25.7667	0.6198	33.4129	0.636	33.2203	0.7966	47.5705
15	0.6347	24.6961	0.6569	32.9101	0.6529	30.9969	0.847	40.7496
20	0.5585	27.5031	0.6815	32.4791	0.626	32.2574	0.8232	44.5112
25	0.5806	26.4324	0.69	31.5903	0.6325	31.3081	0.8506	40.8860
27	0.5525	28.3341	0.6567	32.9130	0.6091	31.8712	0.8431	43.6942
29	0.6472	24.3675	0.6424	33.0869	0.5427	35.5674	0.8019	43.1316
31	0.5551	25.7194	0.636	34.2308	0.4968	34.8036	0.8533	37.4386
33	0.4952	25.5193	0.6862	31.1386	0.539	36.4987	0.8494	41.6286
35	0.5732	26.3342	0.6092	33.6717	0.5479	35.0874	0.8078	47.5894

Overall, Table 4 showed that the prediction performance for tetracycline hydrochloride in paddy field water is the best, with the highest R^2^ of 0.8533 and the lowest R^2^ of 0.7301. The prediction performance for doxycycline hydrochloride was the second best, with the highest R^2^ of 0.69 and the lowest R^2^ of 0.5744. The prediction performance for ofloxacin was worse, with the highest R^2^ of 0.6529 and the lowest R^2^ of 0.4968. Sulfamerazine exhibited the poorest predictive performance, with its coefficient of determination ranging from a maximum of 0.7894 to a minimum of 0.6443, all lower than those of the other three veterinary drugs. Based on the analysis of experimental design and material properties, the core reasons for the low coefficient of determination were as follows: First, sulfamerazine had a weak dielectric response in paddy field water. As a sulfonamide compound, sulfamerazine contained fewer polar functional groups (e.g., amino groups and sulfonamide groups) with lower activity compared to tetracycline veterinary drugs such as doxycycline hyclate and tetracycline hydrochloride, as well as fluoroquinolone veterinary drugs such as ofloxacin. When sulfamerazine dissolved in paddy field water, it caused only slight changes in the dielectric constant and conductivity of the solution. This resulted in weak variations in the amplitude ratio and phase difference signals detected by the interdigital electrodes, which showed poor correlation with concentration gradients, thereby impairing the model’s accuracy in capturing concentration changes. Second, its solubility and molecular structural characteristics led to low detectability of the detection signals. Table 4 presented all prediction results and analyses of the multi-input multi-output model for typical veterinary drugs in paddy field water using amplitude ratio coupled with phase difference data across 10 temperatures.

#### The prediction results and analysis of three models

Each multiple-input multiple-output model had four sets of output data. By integrating these four sets of data, the R^2^ value of the combined dataset was calculated. Table 5 revealed that the content prediction models with amplitude ratio data showed a prediction R^2^ range of 0.7358 to 0.8075 and a minimum root meant square error of prediction (RMSEP) ranged of 26.4966 to 30.6812 mg/L. The content prediction models established with phase difference data demonstrated a prediction R^2^ range of 0.7831 to 0.8713 and an RMSEP ranged of 22.0759 to 28.1526 mg/L. The content prediction models established with amplitude ratio and phase difference data exhibited a prediction R^2^ range of 0.6543 to 0.7651 and an RMSEP ranged of 29.3593 to 35.5591 mg/L. In summary, the content prediction models established with phase difference data achieved the best performance, while those established with amplitude ratio data showed relatively lower performance and the model used amplitude ratio coupled with phase difference data performed the worst. Therefore, a multi-input and multi-output approach was selected to establish a CARS-ANN-based prediction model with phase frequency response data as the input for the prediction of typical veterinary drug content in paddy water in this paper.

**Table 5:** The prediction results and analysis of three models.

Temperature (℃)	Amplitude ratio		Phase difference		Amplitude ratio coupled with phase difference	
	R^2^	RMSEP (mg/L)	R^2^	RMSEP (mg/L)	R^2^	RMSEP (mg/L)
5	0.8075	26.4966	0.8449	24.0407	0.6543	35.5591
10	0.7437	30.6812	0.8339	24.6160	0.7249	31.7011
15	0.8075	26.5944	0.8713	22.0759	0.761	29.8156
20	0.7598	29.6823	0.8356	24.8553	0.7462	30.4538
25	0.8016	26.9140	0.8393	24.2723	0.7651	29.3593
27	0.7583	29.6896	0.829	25.1079	0.7453	30.5362
29	0.7358	30.5222	0.7985	27.1981	0.722	31.9311
31	0.7785	27.7995	0.8139	26.2132	0.7357	31.2459
33	0.7619	29.7856	0.8413	24.0978	0.7363	31.0260
35	0.7513	29.4151	0.7831	28.1526	0.7078	32.7420

It should be noted that the method of dielectric property detection + CARS feature extraction + MIMO-ANN modeling adopted in this study still had certain limitations. First, the discrimination accuracy for veterinary drugs with similar molecular structures and close dielectric parameters was limited, and the generalization ability of the model was affected by the regional differences of paddy field water matrix. Second, the existing electrodes had insufficient ability to capture signals of veterinary drugs with low concentration and weak dielectric response, and the feature screening of the CARS algorithm had slight randomness. Third, the practical application needed to rely on power supply and data transmission modules, which imposed certain constraints on continuous monitoring in remote areas and operation by users without professional background.

However, this method had remarkable scientific and practical significance. From the scientific perspective, it for the first time established a three-dimensional correlation model for the dielectric detection of veterinary drugs in paddy field water, provided a new paradigm for the rapid detection of multi-component pollutants, and verified the adaptability of feature screening algorithms in embedded systems. From the practical perspective, it addressed the pain points of traditional detection methods such as high cost and long time consumption, supported real-time data upload and centralized management, offered a low-cost and high-efficiency technical solution for the monitoring of veterinary drug residues in paddy field water, and thus conformed to the development needs of smart agriculture. Future research will aim at the above limitations and further improve the method system by optimizing electrode structures, introducing transfer learning algorithms, simplifying operation procedures, and other means.

### Industrial application and regulatory implementation

The core components of the sensor (including interdigital electrodes and the STM32F405RGT6 chip) are all mature mass-produced devices. After mass production, the unit cost can be controlled within 2000 RMB. Equipped with engineering designs such as IP67 waterproof rating and solar energy supplementary power supply, the sensor can operate continuously for 7–10 days on a single charge. It supports 24/7 uninterrupted monitoring and cloud-based data management in large-scale paddy fields, which is well-suited for the centralized monitoring needs of agricultural bases. By following the implementation path of “pilot promotion—university-enterprise cooperative mass production—policy alignment”, this sensor can be rapidly integrated into China’s Action Plan for the Development of Smart Agriculture and included in the recommended catalogue of agricultural environmental monitoring equipment.Future research will further optimize the detection accuracy for low concentrations, facilitate the sensor’s acquisition of the EU CE certification and China’s Pattern Approval of Measuring Instruments, and accelerate its widespread deployment in industrial monitoring and regulatory compliance scenarios both domestically and internationally.

## Conclusion

A typical veterinary drug sensor based on interdigitated electrodes was designed in this paper, with the interdigitated electrodes being optimized to select the optimal elec-trode structure. The interdigitated electrodes were used for real-time monitoring of paddy field water, obtaining the content values of sulfamerazine, doxycycline hydrochloride, ofloxacin, and tetracycline hydrochloride, as well as the current water temperature. A microcontroller was employed to collect data information, and a LoRa module was used for wireless transmission to upload the data to an upper computer, enabling real-time monitoring of the soil environment. A multi-input multi-output model for veterinary drugs in paddy field water was established and ported to an STM32 chip. An application for predicting veterinary drug content in paddy field water was designed, allowing the sensor to perform rapid real-time monitoring. The experimental results demonstrate that this sensor exhibits distinct advantages including rapid detection, high portability, pollution-free operation, and suitability for the real-time monitoring of paddy field water. It plays a vital role in improving the visualization and informatization level of paddy field water environment monitoring as well as promoting the development of smart agriculture. Meanwhile, this study still has certain limitations: first, the detection accuracy for low-concentration sulfamethazine needs to be improved; second, the anti-interference capability of the sensor in complex matrices (e.g., paddy field water with high organic matter content and high turbidity) requires further enhancement; third, the model fails to consider the impact of long-term field conditions (such as abrupt temperature changes and rainfall scouring) on detection stability. Future improvement directions will focus on addressing the aforementioned limitations: optimizing the micro-nano structural design of interdigital electrodes to enhance the sensitivity of capturing weak dielectric signals; introducing a multi-feature fusion algorithm to strengthen the anti-interference performance in complex matrices; conducting long-term field experiments to establish a dynamic compensation model for environmental factors, thereby further improving the practical application reliability of the sensor.

## Data Availability

The original contributions presented in this study are included in the article. Further inquiries can be directed to the corresponding author.
